# Partisan Media, Trust, and Media Literacy: Regression Analysis of Predictors of COVID-19 Knowledge

**DOI:** 10.2196/53904

**Published:** 2024-07-24

**Authors:** Kristy Roschke, Alexis M Koskan, Shalini Sivanandam, Jonathan Irby

**Affiliations:** 1 Walter Cronkite School of Journalism and Mass Communication Arizona State University Phoenix, AZ United States; 2 College of Health Solutions Arizona State University Phoenix, AZ United States

**Keywords:** COVID-19, misinformation, media literacy, news consumption, institutional trust, media, trust, prevention, control, health care professional, health care

## Abstract

**Background:**

The COVID-19 pandemic was a devastating public health event that spurred an influx of misinformation. The increase in questionable health content was aided by the speed and scale of digital and social media and certain news agencies’ and politicians’ active dissemination of misinformation about the virus. The popularity of certain COVID-19 myths created confusion about effective health protocols and impacted trust in the health care and government sectors deployed to manage the pandemic.

**Objective:**

This study explored how people’s information habits, their level of institutional trust, the news media outlets they consume and the technologies in which they access it, and their media literacy skills influenced their COVID-19 knowledge.

**Methods:**

We administered a web-based survey using Amazon Mechanical Turk (MTurk) to assess US adults’ (n=1498) COVID-19 knowledge, media and news habits, media literacy skills, and trust in government and health-related institutions. The data were analyzed using a hierarchical linear regression to examine the association between trust, media literacy, news use, and COVID-19 knowledge.

**Results:**

The regression model of demographic variables, political affiliation, trust in institutions, media literacy, and the preference for watching Fox or CNN was statistically significant (R^2^=0.464; *F*_24,1434_=51.653; *P*<.001; adjusted R^2^=0.455) in predicting COVID-19 knowledge scores. People who identified as politically conservative, watched Fox News, and reported lower levels of institutional trust and media literacy, scored lower on COVID-19 knowledge questions than those who identified as politically liberal, did not watch Fox News and reported higher levels of institutional trust and media literacy.

**Conclusions:**

This study suggests that the media outlets people turn to, their trust in institutions, and their perceived degree of agency to discern credible information can impact people’s knowledge of COVID-19, which has potential implications for managing communication in other public health events.

## Introduction

### Background

The global public health crisis caused by COVID-19 dominated the news cycle throughout the pandemic [1]. Because the news media play an essential role in communicating during a public health emergency [2], the public was exposed to a relentless stream of information about the number of cases and deaths, mitigation, and treatment strategies. The overwhelming amount of news on the subject was exacerbated by an influx of false or misleading information about the virus, which caused the World Health Organization to declare a simultaneous “infodemic”—too much information, including misinformation—during a public health event [3,4].

The availability and nature of COVID-19 information changed throughout the pandemic. In 2020, during the earliest months of the pandemic, there was an absence of accurate information about the virus as experts worked to learn more. From the data voids [5] emerged some of the early and persistent COVID-19 misinformation [6]. As experts learned more about the virus, its mitigation, and prevention, and vaccination policies were implemented, misinformation continued evolving on digital and social media platforms to include alternatives to the official messaging. This included content produced by individuals on social media and falsehoods spread by authoritative sources, including some medical professionals and government officials [7,8]. The wide availability of information from sources and platforms of varied credibility left many people unsure of where to find reliable information and contributed to persistent misinformation about the virus [9]. COVID-19 misinformation not only impacted individual information consumers, but it also affected health experts providing guidance. Throughout the pandemic, several pieces of misinformation—including about how the virus is transmitted and how best to treat it—gained traction. The reach and scale of the misinformation affected public health and medical practitioners’ ability to accurately inform the public on treatment [10]. Confusion about proper treatment and prevention coming from the media compounded mistrust of institutional knowledge [11], and as treatment and prevention issues became more politicized, alternate media narratives emerged.

### Prior Work

The connection between what people know or believe about a public health event and where they get their information has been previously studied. Exposure to COVID-19 misinformation has been found to negatively impact COVID-19 knowledge [12]. For example, the Kaiser Family Foundation published survey results in November 2021 reporting that 32% of US adults agreed with at least 1 of 4 incorrect statements about COVID-19, and nearly 80% of respondents believed at least 1 COVID-19 myth [13]. Research has shown that greater knowledge of infectious diseases impacts people’s health-seeking behaviors, which makes the presentation of clear, reliable information a critical component of public health campaigns [14-16].

Studies on news consumption and COVID-19 knowledge have found that all media sources contributed to disseminating both accurate information and misinformation [7]. Concerns about news circulating on social media and exposure to misinformation have been widely researched [17-19], but studies have also found that consumption of certain traditional mainstream news outlets has been associated with belief in misinformation [13]. Other research has found that social media news use, web-based communities, and media literacy may be impacted by the COVID-19 infodemic [20].

### Goal of the Study

The goal of this study is to add to research about misinformation and health by exploring other important variables related to public health and the digital media environment: the public’s trust in relevant major institutions and their levels of media literacy. Given the role mass media played in COVID-19 prevention strategies and vaccine uptake [21], it is important to explore where and how people consume news to better understand which messengers have the most impact on health knowledge. Further, it is critical to understand which institutions individuals trust, as mistrust in science and government is predictive of conspiratorial beliefs [22]. It is also important to assess individuals’ news and media literacy skills in navigating complex information environments [23], which may help mitigate the impact of misinformation [24]. Media literacy informs decision-making and increases individual agency through reflection on media, including critical analysis of a media messages’ meaning and context and assessment of source credibility [25].

Since media literacy coupled with scientific knowledge is associated with preventative behaviors [25], this study aims to explore how people’s information habits, including their level of institutional trust, weekly news diet, and media literacy skills influenced their COVID-19 knowledge. Our research questions (RQs) include the following:

- RQ1: Is there a relationship between people’s information habits and COVID-19 knowledge?
- RQ2: Is there a relationship between institutional trust and COVID-19 knowledge?
- RQ3: Can information habits, institutional trust, and media literacy predict COVID-19 knowledge?

## Methods

### Ethical Considerations

We gained institutional review board approval (STUDY00015977) from the Arizona State University to conduct a web-based survey hosted on QuestionPro (Survey Analytics LLC) to assess US adults’ media habits and their relationship to COVID-19 knowledge. The institutional review board submission included a description of the research and methodology, as well as our method of recruiting participants for the web-based survey through Amazon Mechanical Turk (MTurk). In addition, the consent letter was approved and shared with survey participants prior to beginning the survey, which identified the research team, purpose of the research, estimated survey completion time, compensation rate, and data storage and confidentiality procedures. Survey participants were made aware that their answers and any identifying information shared would remain confidential and would be accessible on the web by the research team via password-protected storage.

### Survey Recruitment

We deployed the survey from September 1-7, 2022, via MTurk, a crowdsourcing platform that has become a popular mechanism for recruiting diverse participants for quantitative research [26]. To improve participant quality, participants were filtered through CloudResearch (Prime Research Solutions, LLC), which screens participants to improve upon overcoming the limitations of basic MTurk sampling [27]. We collected IP addresses to ensure the removal of duplicate responses. After completing the survey, participants were paid (US $1.75) through MTurk.

### Survey Measures

We developed a 58-item survey, adapted from previous relevant research, which included 1 attention check that assessed participants’ COVID-19 knowledge, institutional trust, media literacy, and media consumption. Participants also answered a series of questions about their typical media use, their trusted sources of information, and demographic characteristics (Multimedia Appendix 1).

### Dependent Variable

Our study dependent variable was COVID-19 knowledge, assessed using an 18-question COVID-19 knowledge quiz comprising questions from a previous Kaiser Family Foundation survey [13] and some developed by the research team and reviewed by an external group of subject matter experts (Multimedia Appendix 2). The total knowledge score was calculated by assigning 1 point to every correct answer for a total score of 18.

### Independent Variables

Study independent variables included demographic information, institutional trust, media habits, and self-perceptions of their media literacy. Sociodemographic factors included sex, race, ethnicity, age, community of residence, and education level. To measure trust, participants answered 6 questions assessing their trust in different entities charged with disseminating COVID-19 knowledge, ranging from federal and state governments to pharmaceutical companies and personal doctors [28]. Trust was measured on a 5-point Likert scale ranging from 1=very low to 5=very high. Participants were also asked about their political affiliation.

Participants were asked several questions about their media habits using survey questions adapted from the Reuters Institute Digital News Report [29]. Participants were asked to select which media outlets they had consumed in the past week from a list of popular choices, naming their main source of news (1=television, 2=newspapers, 3=news websites, 4=radio, 5=podcasts, 6=social media, 7=apps that feature articles from multiple sources, and 8=conversations with others). Participants were also asked which social media platforms they used to consume or share news (Facebook, Twitter, Instagram, YouTube, TikTok, Reddit, WhatsApp, Snapchat, LinkedIn, Telegram, and none) and whether they engaged with news via activities such as rating, liking or favoriting a news story, commenting on a news story, and sharing a news story.

To measure participants’ media literacy, we asked them to rate their level of agreement with several 5-point Likert-type scale questions with response options ranging from 1=strongly disagree to 5=strongly agree. Questions included “I do not like to have to do a lot of thinking (reverse coded),” “If I pay attention to different sources of news, I can avoid being misinformed,” and “I have the skills to interpret media messages.” Questions map to previously validated media literacy constructs described in research, including automatic versus higher-order thinking, the acceptance of information at face value versus engaging in critical thinking about the information; media locus of control, the extent to which people believe they have control over their media use; and self-perceived media literacy, the belief in one’s ability to determine credible information from misinformation [30,31].

### Data Analysis

We conducted all analyses using SPSS (version 28.0.1.1; IBM Corp). All variables were reported in numbers, means, and SDs. For some sociodemographic factors, smaller categories were combined for more meaningful analysis. The COVID-19 knowledge score was used as the dependent variable, calculated as the sum of all correct answers on the 18-point COVID-19 knowledge questions. We conducted a hierarchical linear regression to test the association between trust, media literacy, news use, and COVID-19 knowledge scores. This test was used in order to determine the percent of variance attributed to certain variables separately from one another.

## Results

### Overview

A total of 1573 people responded to the survey, with a completion rate of 96% (n=1510); 12 were removed due to missing answers for a final sample of 1498 US adults. The majority of the sample was female participants (n=776, 51.8%) and White (n=1134, 75.7%). The median age of the sample was 40 (IQR 33-53) years. Descriptive statistics of all demographic variables are listed in Table 1.

**Table 1:** Demographic information from the web-based survey (n=1498) of COVID-19 knowledge, news and information habits, institutional trust, and media literacy. The survey sample was 51.8% (n=776) female participants; 59% (n=884) between the ages of 18-44 years; 37.5% (n=561) from suburban areas; and 75.7% (n=1134) White.

Variable			Values, n (%)
**Sex**			
	Male	697 (46.5)	
	Female	776 (51.8)	
	Other or prefer not to say	14 (0.9)	
**Age range per Medicaid (years)**			
	18-44	884 (59)	
	45-64	480 (32)	
	≥65	119 (7.9)	
**Community**			
	Rural	232 (15.5)	
	Small city or town	425 (28.4)	
	Suburb near large city	561 (37.4)	
	Large city	276 (18.4)	
**Race or ethnicity**			
	Asian	109 (7.3)	
	Black or African American	129 (8.6)	
	White	1134 (75.7)	
	Hispanic or Latino	69 (4.6)	
	Other or prefer not to say	57 (3.8)	
**Education, n (%)**			
	High school or lower	156 (10.4)	
	Some college	312 (20.8)	
	Vocational or college degree	723 (48.3)	
	Some graduate school or graduate degree	307 (20.5)	
**Political affiliation**			
	Democrat	671 (44.8)	
	Moderate	106 (7.1)	
	Republican	341 (22.8)	
	Independent, other, prefer not to say	377 (25.2)	

### Dependent Variable

Nearly 60% (n=864) of respondents scored either 17 or 18 on the 18-point knowledge quiz, indicating that most people at the time of the survey had a basic understanding of the most common facets of the COVID-19 virus and preventative measures. Table 2 illustrates the distribution of correctly answered COVID-19 knowledge questions.

**Table 2:** Distribution of scores on the 18-question COVID-19 knowledge test (n=1498). Nearly 60% (n=864) of survey respondents earned a perfect score of 18/18 or a near-perfect score of 17/18.

Score on COVID-19 knowledge quiz	Respondents earning this score, n (%)
18	603 (40.3)
17	261 (17.4)
15	127 (8.5)
14	86 (5.7)
13	65 (4.3)
12	62 (4.1)
11	56 (3.7)
10	44 (2.9)
9	48 (3.2)
8	45 (3)
7	22 (1.5)
6	17 (1.1)
5	4 (0.3)
4	2 (0.1)
3	1 (0.1)
2	0 (0)
1	0 (0)

### Independent Variables

Before conducting the regression analyses, we reduced the number of items we would enter into the various models. Overall, participants reported neutral scores for their trust in institutions and health-related entities (eg, government, pharmaceutical companies, and health care providers) connected with COVID-19 prevention and control. The mean composite score of institutional trust across all entities was 3.13 (SD 0.87; 1=very low and 5=very high). Taken separately, mean trust scores were higher for doctors (mean 3.68, SD 0.96) than the other entities included in the individual questions (other scores: national government: mean 2.96, SD 1.13; state government: mean 2.95, SD 1.09; CDC: mean 3.18, SD 1.26; pharmaceutical companies: mean 2.55, SD 1.04; and pharmacist: 3.46, SD 0.94). Using SPSS, we conducted a correlation analysis on the individual trust variables and found responses to be highly correlated (α=.895), so items were combined into 1 overall trust variable, and the mean score for each participant was used in the analysis.

Additionally, a principal components analysis was conducted to reduce the media literacy dimensions into fewer dimensions, in accordance with the research [30,31]. The correlation matrix indicated that all variables had at least 1 correlation coefficient greater than 0.3. The Kaiser-Meyer-Olkin measure was 0.7, with individual Kaiser-Meyer-Olkin measures all greater than 0.6, which are classifications of “middling” to “meritorious” according to Kaiser [32]. Bartlett test of sphericity was statistically significant (*P*<.001), indicating that the data were likely factorizable. The 3 factors that emerged with eigenvalues greater than 1 are consistent with the literature: automatic versus higher-order thinking, media locus of control, and self-perceived media literacy (percentage variance explained=37.4%, 22.9%, and 13%, respectively). The composite score created by the SPSS calculation was used in the analysis.

In the first block of the hierarchical linear regression, we controlled for certain demographic characteristics, including age, race or ethnicity, education, and community of residence. Because prior research has shown political affiliation and institutional trust to have an impact on COVID-19 knowledge [11], these variables were entered in separate blocks of the regression. We then entered separate blocks in the model for the 3 media literacy composite factors and media outlets viewed in the past week. Though we asked survey participants about their recent consumption across 21 media outlets and platforms, only a handful were visited by more than 20% (n=302) of the sample. We included only Fox News (and the Fox News website) and CNN (and the CNN website) in the final model of the regression analysis because of their relatively high response rate in the survey and their recognition as partisan media [29].

The full hierarchical regression model of the demographic variables, political affiliation, trust in institutions, media literacy, and the preference for watching Fox or CNN was statistically significant (R^2^=0.464; *F*_24,1434_=51.653; *P*<.001; adjusted R^2^=0.455) in predicting the COVID-19 knowledge scores. Table 3 illustrates the outcomes of the regression, showing that many, but not all, of the participants’ characteristics and news preferences correlated with perfect and near-perfect COVID-19 knowledge scores.

**Table 3:** Results from the hierarchical regression indicating the predictors of scoring perfect (18/18) or near-perfect (17/18) on the COVID-19 knowledge test. Significant positive predictors of a high score on the COVID-19 knowledge test include several demographic factors, a higher media locus of control and institutional trust composite score, and being a recent consumer of CNN website content. Significant negative predictors include aligning politically as a moderate, Republican, or independent and being a recent watcher of Fox News^a^.

Predictors			β	SE	β	*P* value
**First block**						
	Age		.016	0.007	.065	.02
	**Community**					
		Small city or town	1.082	0.274	.147	<.001
		Suburb	1.336	0.263	.195	<.001
		Large city	1.368	0.306	.160	<.001
	**Race or ethnicity**					
		Asian	1.104	0.336	.087	.001
		Black or African American	–.992	0.315	–.083	.002
		Hispanic or Latino	–.370	0.416	–.023	.37
		Other or prefer not to say	.197	0.458	.011	.67
	**Sex**					
		Female	.313	0.174	.047	.07
		Other or prefer not to say	1.282	0.884	.038	.15
	**Education**					
		Some college	.352	0.323	.043	.27
		Vocational school or college degree	.529	0.293	.080	.07
		Some graduate school or graduate degree	.733	0.329	.089	.03
**Second block**						
	**Political affiliation**					
		Moderate	–1.158	0.306	–.90	<.001
		Republican	–3.806	0.199	–.481	<.001
		Independent, other, or prefer not to say	–2.068	0.193	–.270	<.001
**Third block**						
	Trust composite score		1.873	0.085	.491	<.001
**Fourth block**						
	Automatic versus mindful processing		.188	0.066	.057	.005
	Media locus of control		.261	0.067	.078	<.001
	Self-perceived media literacy		–.067	0.067	–.020	.32
**Fifth block**						
	Fox News		–1.030	0.190	–.131	<.001
	CNN		.108	0.159	.015	.49
	Fox News website		.057	0.210	.006	.79
	CNN website		.380	0.165	.052	.02

^a^Model 1: *F*_13,1445_=5.799; *P*<.001; adjusted R^2^=.041; model 2: *F*_3,1442_=128.519; *P*<.001; adjusted R^2^=0.242; model 3: *F*_1,1441_=485.440; *P*<.001; adjusted R^2^=0.432; model 4: *F*_3,1438_=8.104; *P*<.001; adjusted *R*^2^=0.441; and model 5: *F*_4,1434_=10.210; *P*<.001; adjusted *R*^2^=0.455.

Specifically, the results of the hierarchical regression analysis revealed significant associations between the demographic factors of community of residence, race, and political affiliation and COVID-19 knowledge scores. Participants residing in urban and suburban areas and small towns tended to have higher levels of COVID-19 knowledge compared to those in rural areas (β=1.368; SE=0.306; *P*<.001; β=1.336; SE=0.263; *P*<.001; and β=1.082; SE=0.274; *P*<.001, respectively). Race and ethnicity were also significant factors. Compared to White respondents, Black or African American participants were more likely to have lower knowledge scores (β=–1.102; SE=0.240; *P*<.001), and Asian participants were more likely to have higher knowledge scores (β=–.992; SE=0.315; *P*=.002 and β=1.104; SE=0.336; *P*=.001, respectively). Age and education were also significant but with a small coefficient.

Our results show that political affiliation and partisan media use, levels of institutional trust, and media literacy all have a significant relationship to COVID-19 knowledge. Political affiliation and the trust in institutions composite score collectively accounted for 38.9% of the variance in COVID-19 knowledge, with R^2^ change of 0.201 and 0.189, respectively. Concerning political affiliation, Republicans and the category of independents, other, and those who selected “prefer not to say” had lower COVID-19 knowledge scores than Democrats (β=–1.731; SE=0.201; *P*<.001 and β=–0.714; SE=0.175; *P*<.001, respectively). The composite score of institutional trust in entities like federal and state government, pharmaceutical companies, doctors, and pharmacists was also statistically significant in the final model, with higher levels of trust associated with higher knowledge scores (β=1.784; SE=0.086; *P*<.001). The media literacy factors of automatic versus mindful processing and media locus of control, which assess an individual’s propensity to engage in critical thinking and to feel in control of the media they consume, respectively, were both significant in the model; however, they account for very little of the variance. Self-perceived media literacy did not significantly correlate with COVID-19 knowledge.

Finally, Fox News viewership and CNN website readership were both significant in the final model, though the Fox News website and CNN were not. Fox News viewership has a greater impact on COVID-19 knowledge than the CNN website with Fox watchers having lower knowledge scores than non-Fox watchers (β=–1.030; SE=0.190; *P*<.001 and β=.380; SE=0.165; *P*=.02, respectively).

## Discussion

### Principal Findings

This study found significant relationships between people’s levels of institutional trust, news habits, and media literacy skills and their COVID-19 knowledge. Study findings confirmed that institutional trust in science and various levels of the government was positively correlated with COVID-19 knowledge scores. As a contribution to misinformation research, this study identified that aspects of media literacy—mindful processing and media locus of control— predicted higher levels of COVID-19 knowledge. This study also confirmed that it matters where individuals receive their news information, with more participants who watched CNN having higher levels of COVID-19 knowledge than their Fox News–watching counterparts [8].

Demographic variables in our model that predicted COVID-19 knowledge and trust in expert health information are consistent with characteristics that have emerged in other research: community, race or ethnicity, education, and political affiliation [33,34]. Additionally, the results of this study show that political affiliation and the partisan information environment impacted COVID-19 knowledge. Participants who identified as Democrat and who had higher levels of trust in institutional actors, such as federal and state governments, the Centers for Disease Control and Prevention, pharmaceutical companies, and doctors, scored higher on the COVID-19 knowledge test, with these 2 variables accounting for 39% of the variance in the final regression model. This aligns with past research that reported Republicans’ declining trust in scientists and medical researchers since the beginning of the pandemic [35]. One important area for future research would be to test targeted health messaging [36] to different audiences based on their reported political affiliation and trust in certain institutions, in addition to other cultural and demographic factors traditionally considered by public health officials [37].

This study found that participants used web-based sources for their news information. Research has shown that people across generations have trouble discerning credible news on digital and social media [38]. As digital and social media have become the most popular places for people to view news, concern about misinformation on these platforms has risen to peak levels [39]. These characteristics of modern information ecosystems became even greater challenges during the real-time events of the pandemic, as people looked to a wide variety of information sources to stay informed.

Research has focused on the influence of social media on health misinformation [37]. Though most survey respondents indicated they used social media at least sometimes for news (n=898, 59.9%)—more than any other single media distribution channel included in the survey—social media use was not a significant predictor of COVID-19 knowledge in our model. This may be because the category “social media” could include a variety of different platforms (such as Twitter, Facebook, YouTube, and TikTok) used by a broad cross-section of people. Therefore, the term “social media” may have been too general to make a significant impact on the model. However, even though it did not significantly impact COVID-19 knowledge, there is an opportunity to further investigate the relationship between social media use, partisanship, and trust to assess its role in spreading health information—good and bad.

Notably, also significant in the model was the participants’ level of media literacy, with 2 of the 3 media literacy variables (automatic vs mindful processing and media locus of control) having a positive association with COVID-19 knowledge. Though the effect on the overall model was small, the inclusion of media literacy variables represents a novel contribution to the association between knowledge and media habits. Our findings suggest that the extent to which people feel in control of their media use—including the places they seek news and the information they receive and choose—and their confidence in exercising critical thinking skills to discern credible information from a variety of sources positively impact knowledge. Self-perceived media literacy was not significant in the model, which may be because people tend to inflate their media literacy skills when compared with their actual practices [31]. Given the deluge of information people were exposed to during the pandemic, it is key that those who participate in more deliberate media use are more likely to recognize COVID-19 myths and misinformation. This indicates that greater access to media literacy education and the development of targeted informational campaigns from trusted messengers can play a critical role in disseminating health information.

Finally, the recent consumption of 2 widely known and popular US cable news channels, Fox News and CNN, was associated with COVID-19 knowledge, though in different ways. Fox News broadcast viewership was negatively related to COVID-19 knowledge, while CNN did not significantly impact score. This finding also aligns with previous research on COVID-19 misinformation, particularly when taken with political affiliation and trust. Additionally, studies have shown that Fox News viewership is associated with lower adoption of COVID-19 prevention behaviors such as social distancing, and increased engagement in behaviors is considered risky during the early stages of the virus, such as going to places with more than 10 people [8,40]. Interestingly, the CNN website viewership was positively related to COVID-19 knowledge, though the Fox News website was not statistically significant in the final model. One possible explanation for the websites of the major broadcast cable outlets performing differently in the model is that the television or video experience, which prominently features bombastic pundits and personalities, is more outwardly opinion-based and potentially more persuasive to certain audiences than the text-dominated websites that appear more muted in tone. Another area of future research would be to examine if people perceive cable news as serving more of an entertainment or informational purpose and how that relates to people’s knowledge of current health information.

### Limitations

Survey data were drawn from a web-based panel, which does not represent a random sample of all US adults, a common problem in research that relies on web-based surveys. For example, this study oversampled White populations as well as Democrats and highly educated people, limiting the extent to which we can generalize the findings. However, this method of recruitment allowed our team to sample a large, somewhat diverse sample of US adults*.* Additionally, with the survey, we are relying upon respondents to provide an accurate recall of their news use, which can be difficult to do [41]. However, the study’s findings are in line with previous research on the topic and thus add to the collective knowledge of the impact of COVID-19–related media use and misinformation.

Finally, we conducted this survey at a time when several policies about key mitigation strategies like social distancing and mask-wearing were no longer mandated but still in use in many places. People’s knowledge and media consumption about COVID-19 likely changed as the pandemic progressed, so this study should be considered within that context. It may be useful to conduct a follow-up study now that the United States has ended the national emergency [42], to see if knowledge has changed, which can have potential implications on public health campaigns in a future outbreak.

### Conclusions

COVID-19 was a global pandemic that reflected the modern times in which it occurred; the dissemination of information about the virus and the prevalence of misinformation were impacted by the existing digital and social media environment. Just as the virus spread rapidly because of the effects of globalization, so, too, did information about the virus spread and evolve on a global scale. Crisis events such as the COVID-19 pandemic highlight the importance of good information habits and underscore the need for better media literacy as part of the public health response.

This study’s survey results support previous research on the role of partisan media in spreading misinformation and contributes additional findings by connecting it to participants’ media literacy. Two factors related to media literacy, mindful processing of media information and an individual’s locus of control, had a significant positive impact on finding reliable COVID-19 information in mediated environments. As the digital information environment is ever-evolving and the threat of misinformation continues to loom, this finding suggests a need for greater media literacy education for people of all ages. Public health officials can play a role by considering media literacy in their health outreach and incorporating media literacy messages in their health campaigns.

Access to and acceptance of credible health information are key to successful health communication and educational campaigns and other health interventions. This study found that the information environment during a high-impact, highly publicized health event can be shaped by partisan beliefs and their relative impact on institutional trust and media selection.

## Appendix

Multimedia Appendix 1 Complete survey instrument.

Multimedia Appendix 2 List of COVID-19 knowledge questions asked in the web-based survey.

## Abbreviations

MTurk: Amazon Mechanical Turk
RQ: research question

## Data Availability

The data sets generated and analyzed during this study are available from the corresponding author on reasonable request.
