# Supplementary material for: Partisan Media, Trust, and Media Literacy: Regression Analysis of Predictors of COVID-19 Knowledge
Source: JMIR Form Res. 2024 Jul 24;8:e53904. doi: 10.2196/53904 (PMC11306951; doi:10.2196/53904)
Supplement: Multimedia Appendix 1 [file formative_v8i1e53904_app1.pdf]

Based on your responses to this survey, a researcher may follow up with you for a one-time phone interview for $25. If you are interested in being interviewed, please provide an email address where we can reach you.

Please add your Worker ID

How would you describe your gender?

- ◯ Male
- ◯ Female
- ◯ Other

What is your age?

What languages are spoken in your home? Mark all that apply.

- ☐ English
- ☐ Spanish
- ☐ Other

What type of community do you live in?

- ◯ Rural area
- ◯ Small city or town

QuestionPro

- Suburb near a large city
- Large city

Which of the following best describes you?

- Hispanic or Latino
- American Indian or Alaska Native
- Asian
- Black or African American
- Native Hawaiian or Other Pacific Islander
- Caucasian or White
- Multiracial
- Prefer not to say
- Other

Are you Hispanic or Latino?

- Yes
- No

What is the highest level of education you have completed?

- Some high school
- High school graduate
- Some college
- Ttrade/technical/vocational training

- College graduate
- Some postgraduate work
- Post-graduate degree

How would you describe your current employment status?

- Full-time
- Part-time
- Not employed
- Retired
- Unable to work
- Other

What was your total household income last year?

- $0-25,999
- $26,000-$51,999
- $52,000-74,999
- $75,000 - $99,999
- $100,000 – 200,000
- More than $200,000
- Do not know/prefer not to say

We hear a lot of talk these days about the influence of politics on COVID-19 protective behaviors. Where would you place yourself among the following categories?

- Democrat

- Moderate
- Republican
- Independent
- Prefer not to say
- Other

What level of trust do you have in the information about COVID-19 from your national government?

- Very low
- Low
- Moderate
- High
- Very high

What level of trust do you have in the information about COVID-19 from your state/territorial government?

- Very low
- Low
- Moderate
- High
- Very high

What level of trust do you have in the information about COVID-19 coming from the Centers for Disease Control and Prevention (CDC)?

- Very low

QuestionPro

- Low
- Moderate
- High
- Very high

What level of trust do you have in pharmaceutical companies?

- Very low
- Low
- Moderate
- High
- Very high

What level of trust do you have in your doctor?

- Very low
- Low
- Moderate
- High
- Very high

What level of trust do you have in your pharmacist?

- Very low
- Low
- Moderate
- High

- ◯ Very high

Are you covered by any kind of health insurance or some other kind of health care plan?

- ◯ Yes
- ◯ No

Do you currently have a primary care provider?

- ◯ Yes
- ◯ No

Have you previously been diagnosed with COVID-19?

- ◯ Yes
- ◯ No

Have you known someone who has died due to complications with COVID-19?

- ◯ Yes
- ◯ No

Have you or someone you know ever been hospitalized due to complications with COVID-19?

- ◯ Yes
- ◯ No

Have you vaccinated against COVID-19?

- ◯ Yes, I received all doses and 1 or more booster doses (1 shot for J&J, 2 for Pfizer & Moderna)

- ◯ Yes, I received the original doses but no booster dose (1 shot for J&J, 2 for Pfizer/Moderna)
- ◯ No, but I plan on vaccinating
- ◯ No, and I do not plan on vaccinating

COVID-19 vaccines (Pfizer, Moderna, J&J) are safe for most recipients.

- ◯ True
- ◯ False

COVID-19 vaccines (Pfizer, Moderna, J&J) are effective in preventing hospitalization.

- ◯ True
- ◯ False

COVID-19 vaccines (Pfizer, Moderna, J&J) are effective in preventing death from complication with the virus.

- ◯ True
- ◯ False

Regular use of masks in high-risk settings will lessen someone's risk of developing or spreading COVID-19.

- ◯ True
- ◯ False

Avoiding close contact with others who have been exposed to or are sick with COVID is a key strategy in preventing COVID-19.

- ◯ True

False

Those who are fully vaccinated cannot transmit COVID-19.

True

False

The use of masks in schools has not shown to reduce the risk of COVID-19 infection in children.

True

False

COVID-19 can only be spread by those who are exhibiting symptoms.

True

False

Those who have had COVID-19 cannot get the virus again.

True

False

Social distancing (staying 6 feet away from others) can help prevent the spread of COVID.

True

False

The government is exaggerating the number of COVID-19 deaths.

True

False

Pregnant women should not get the COVID-19 vaccine.

- True
- False

COVID-19 vaccines have been shown to cause infertility.

- True
- False

You can get COVID-19 from the vaccine.

- True
- False

COVID-19 vaccines can change your DNA.

- True
- False

People of all ages can become infected with COVID-19.

- True
- False

People of all racial and ethnic groups can become infected with COVID-19.

- True

◯ False

Most people who are infected with the COVID-19 virus recover from it.

◯ True

◯ False

Please tell me how much you agree or disagree with each statement.

	Strongly Disagree	Disagree	Neutral	Agree	Strongly Agree
I do not like to have to do a lot of thinking.	◉	◯	◯	◯	◯
I try to avoid situations that require thinking in-depth about something.	◉	◯	◯	◯	◯
I prefer complex to simple problems.	◉	◯	◯	◯	◯
I am in control of how I receive news (e.g., which newspapers, TV shows, websites I visit).	◉	◯	◯	◯	◯
When I am misinformed by the news media, I am to blame.	◉	◯	◯	◯	◯
If I pay attention to different sources of news, I can avoid being misinformed.	◉	◯	◯	◯	◯
If I take the right actions, I can stay informed.	◉	◯	◯	◯	◯

Most people think the news has ...

◯ a greater effect on themselves than other people.

◯ a greater effect on other people than themselves.

◯ the same effect on themselves as others.

◯ does not have any effects on anyone.

◯ do not know.

Please tell me how much you agree or disagree with each statement.

	Strongly disagree	Disagree	Neutral	Agree	Strongly agree
I have the skills to interpret media messages.	◯	◯	◯	◯	◯
I am confident in my ability to judge the accuracy of news.	◯	◯	◯	◯	◯
People should accept information from the news on face value.	◯	◯	◯	◯	◯
It is the role of the press to represent diverse viewpoints.	◯	◯	◯	◯	◯
It is the job of citizens to overcome their biases when consuming news.	◯	◯	◯	◯	◯
People need to review news content critically.	◯	◯	◯	◯	◯

Typically, how often do you access news? By news, we mean national, international, regional/local news and other topical events accessed via any platform (radio, TV, newspaper or online)

◯ More than 5 times a day

◯ Between 2 and 5 times a day

◯ Once a day

◯ 4-6 days a week

◯ 2-3 days a week

◯ Once a week

◯ Less often than once a week

◯ Never

◯ Don't know

How interested are you in politics?

- ◯ Extremely interested
- ◯ Very interested
- ◯ Somewhat interested
- ◯ Not very interested
- ◯ Not at all interested
- ◯ Don't know

How often do you eat cement?

- ◯ Daily
- ◯ Weekly
- ◯ Monthly
- ◯ Yearly
- ◯ Never

Which of the following television news programs have you watched in the last week as a source of news?

- ☐ NBC Nightly News
- ☐ The Today Show
- ☐ Meet the Press
- ☐ PBS Newshour
- ☐ Fox News
- ☐ CNN
- ☐ MSNBC
- ☐ None of these

QuestionPro

- [ ] Other

As a source of news, which of the following newspapers/digital platforms have you read in the last week?

- [ ] The New York Times
- [ ] USA Today
- [ ] Washington Post
- [ ] Wall Street Journal
- [ ] Huffington Post
- [ ] BuzzFeed
- [ ] CNN.com
- [ ] Foxnews.com
- [ ] None of these
- [ ] Other

As a source of news, which of the apps/websites that include news from multiples sources have you read in the last week?

- [ ] Apple News
- [ ] Google News
- [ ] MSN
- [ ] Yahoo
- [ ] Flipboard
- [ ] Social media such as Facebook, Twitter, YouTube, Reddit
- [ ] None of these

☐ Other

Which would you say is your MAIN source of news?

○ Television

○ Newspapers

○ News websites

○ Radio

○ Podcasts

○ Social Media

○ Apps that feature articles from multiple sources (e.g., Apple News, Flipboard)

○ Conversations with others

Please indicate your level of agreement with the following statement.
“Thinking about online news, I am concerned about what is real and what is fake.”

○ Strongly disagree

○ Disagree

○ Neither agree nor disagree

○ Agree

○ Strongly agree

Thinking specifically about coronavirus (COVID-19) and its effects, which of the following online sources do you believe spreads the most false or misleading information?

○ The government, politicians or political parties in my country

○ Foreign governments, politicians or political parties

- ◯ Ordinary people
- ◯ Activists or activist groups
- ◯ Celebrities (e.g. actors, musicians, sports stars)
- ◯ Journalists or news organizations
- ◯ I am not concerned about any of these
- ◯ Don’t know

Thinking specifically about Coronavirus (COVID-19) and its effects, which of the following online sources do you believe spreads the most false or misleading information?

- ◯ News websites or apps
- ◯ Search engines (e.g. Google, Bing)
- ◯ Facebook
- ◯ Twitter
- ◯ YouTube
- ◯ Messaging applications (e.g. WhatsApp, Facebook Messenger)
- ◯ I am not concerned about any of these
- ◯ Don’t know

Thinking specifically about Coronavirus (COVID-19) and its effects, which of the following online sources do you trust most?

- ◯ News websites or apps
- ◯ Search engines (e.g. Google, Bing)
- ◯ Facebook
- ◯ Twitter

- YouTube
- Messaging applications (e.g. WhatsApp, Facebook Messenger)
- I am not concerned about any of these
- Don’t know

Which of the following have you used for sharing or discussing news in the last week? Please select all that apply.

- [ ] Facebook
- [ ] Twitter
- [ ] Instagram
- [ ] YouTube
- [ ] TikTok
- [ ] Reddit
- [ ] WhatsApp
- [ ] Snapchat
- [ ] LinkedIn
- [ ] Telegram
- [ ] None (Did not share or discuss news online)

During an average week, which of the following ways do you share or discuss news coverage? Please select all that apply.

- [ ] Rate, like, or favorite a news story
- [ ] Comment on a news story
- [ ] Share a news story

- [ ] Vote in an online poll via a news site or social network
- [ ] Talk online with friends and colleagues about a news story (e.g. by email, social media, messaging app)
- [ ] Talk with friends and colleagues about a news story (face to face)
- [ ] None of these

What is the best way to locate accurate information about health and disease prevention?

Privacy
