# Supplementary material for: Partisan Media, Trust, and Media Literacy: Regression Analysis of Predictors of COVID-19 Knowledge
Source: JMIR Form Res. 2024 Jul 24;8:e53904. doi: 10.2196/53904 (PMC11306951; doi:10.2196/53904)
Supplement: Multimedia Appendix 2 [file formative_v8i1e53904_app2.docx]

**Multimedia Appendix 2.** List of COVID-19 knowledge questions asked in the web-based survey.

| **Question** | **Correct Response** |
| --- | --- |
|  |  |
| COVID-19 vaccines (Pfizer, Moderna) are safe for most recipients | True |
| COVID-19 vaccines (Pfizer, Moderna) are effective in preventing hospitalization | True |
| COVID-19 vaccines (Pfizer, Moderna) are effective in preventing death. | True |
| Regular use of masks in high-risk settings will lessen your SOMEONE’S risk of developing or spreading COVID-19 | True |
| Avoiding close contact with others who have been exposed to or are sick with COVID is a key strategy in preventing COVID-19 | True |
| Those who are fully vaccinated cannot transmit COVID-19 | False |
| The use of masks in schools has not been shown to reduce the risk of COVID-19 infection in children | False |
| COVID-19 can only be spread by those who are exhibiting symptoms | False |
| Those who have had COVID-19 cannot contract the disease again. | False |
| Social distancing (6 feet away from others) can help prevent the spread of COVID. | True |
| The government is exaggerating the number of COVID-19 deaths. | False |
| Pregnant women should not get the COVID-19 vaccine. | False |
| COVID-19 vaccines have been shown to cause infertility. | False |
| You can get COVID-19 from the vaccine. | False |
| COVID-19 vaccines can change your DNA | False |
| People of all ages can become infected with COVID-19. | True |
| People of all racial and ethnic groups can become infected with COVID-19. | True |
| Most people who are infected with the COVID-19 virus recover from it. | True |
